# Equilibrium-Phase High Spatial Resolution Contrast-Enhanced MR Angiography at 1.5T in Preoperative Imaging for Perforator Flap Breast Reconstruction

**DOI:** 10.1371/journal.pone.0071286

**Published:** 2013-08-29

**Authors:** Bas Versluis, Stefania Tuinder, Carla Boetes, René Van Der Hulst, Arno Lataster, Tom Van Mulken, Joachim Wildberger, Michiel de Haan, Tim Leiner

**Affiliations:** 1 Department of Radiology, Maastricht University Medical Center, Maastricht, The Netherlands; 2 Department of Plastic and Reconstructive Surgery, Maastricht University Medical Center, Maastricht, The Netherlands; 3 Cardiovascular Research Institute Maastricht, Maastricht University Medical Center, Maastricht, The Netherlands; 4 Department of Anatomy and Embryology, Maastricht University, Maastricht, The Netherlands; 5 Department of Radiology, Utrecht University Medical Center, Utrecht, The Netherlands; Cornell University, United States of America

## Abstract

**Objectives:**

The aim was (i) to evaluate the accuracy of equilibrium-phase high spatial resolution (EP) contrast-enhanced magnetic resonance angiography (CE-MRA) at 1.5T using a blood pool contrast agent for the preoperative evaluation of deep inferior epigastric artery perforator branches (DIEP), and (ii) to compare image quality with conventional first-pass CE-MRA.

**Methods:**

Twenty-three consecutive patients were included. All patients underwent preoperative CE-MRA to determine quality and location of DIEP. First-pass imaging after a single bolus injection of 10 mL gadofosveset trisodium was followed by EP imaging. MRA data were compared to intra-operative findings, which served as the reference standard.

**Results:**

There was 100% agreement between EP CE-MRA and surgical findings in identifying the single best perforator branch. All EP acquisitions were of diagnostic quality, whereas in 10 patients the quality of the first-pass acquisition was qualified as non-diagnostic. Both signal- and contrast-to-noise ratios were significantly higher for EP imaging in comparison with first-pass acquisitions (p<0.01).

**Conclusions:**

EP CE-MRA of DIEP in the preoperative evaluation of patients undergoing a breast reconstruction procedure is highly accurate in identifying the single best perforator branch at 1.5Tesla (T). Besides accuracy, image quality of EP imaging proved superior to conventional first-pass CE-MRA.

## Introduction

The number of (prophylactic) mastectomies in (the prevention of) breast cancer is increasing, and so is the number of patients that opt for reconstructive breast surgery after mastectomy [1], [2], [3]. Over the last decade, deep inferior epigastric perforator (DIEP) flap procedures have gained considerable support among plastic surgeons as preferred technique for breast reconstruction [4]. In contrast to the more conventional transverse rectus abdominis musculocutaneous (TRAM) flap procedure, the DIEP flap procedure uses only subcutaneous abdominal fat, centered around the best single large perforator branch of the deep inferior epigastric artery (DIEA) for the blood supply of the flap. Well-known advantages of perforator flaps include less postoperative pain, less donor site complications and less functional impairment compared to TRAM flaps [5], [6]. Disadvantages of the DIEP procedure, on the other hand, include difficulties in harvesting the flap, resulting in considerably longer dissection times, and the fact that long-term results depend heavily on the quality of the perforator branch supplying the flap.

Preoperative evaluation of the DIEA perforator branches in the abdomen to identify adequate perforator branches facilitates surgical planning of the procedure and shortens dissection times [6], [7], [8], [9], [10]. Currently, the most widely applied techniques in preoperative imaging and planning in DIEP flap procedures are Doppler ultrasound (DUS) and computed tomography angiography (CTA) [5]. Doppler, however, is associated with long imaging times, low accuracy and high interobserver variability [5], [11]. CTA, on the other hand, is highly accurate in demonstrating location, size and course of the perforators, but suffers from exposure to ionizing radiation, which is an important drawback in the often (relatively) young patients [12], [13], [14]. Recently, several authors have demonstrated that MR angiography can also be used in preoperative imaging of the perforator branches of the DIEA [5], [6], [8], [15], [16]. Excellent soft-tissue contrast and the absence of ionizing radiation are important advantages of MRI. Nevertheless, experience with contrast-enhanced MR angiography (CE-MRA) in the preoperative workup of patients undergoing DIEP flap procedures is still scarce. Several studies have been performed using state-of-the-art 3T hardware, instead of the more widely available 1.5T magnetic resonance angiography (MRI) systems [5], [6], [8], [15], [16], and most of these studies have employed conventional extracellular contrast agents in combination with first-pass imaging to visualize DIEA perforator branches. Considering the small size of DIEA perforator branches we wondered whether it was possible to obtain high spatial resolution equilibrium-phase (EP) images with improved resolution compared to first-pass acquisitions using a recently described new intravascular contrast agent, gadofosveset trisodium [17], [18]. Blood pool agents have important benefits over conventional small-sized extracellular agents in CE-MRA, such as the lengthened imaging window and the relatively large R1 [19], both allowing longer acquisition times, enabling data acquisition at a very high resolution and with very high accuracy.

The aims of the current study were (i) to investigate the accuracy of equilibrium-phase high spatial resolution CE-MRA at 1.5T using a blood pool contrast agent in the preoperative evaluation of the DIEA perforator branches, and (ii) to compare image quality of equilibrium-phase high spatial resolution imaging with conventional first-pass CE-MRA.

## Material and Methods

### Subjects

Twenty-three consecutive patients (all female, 48.1±9.7 years) scheduled to undergo 36 free flap procedures for breast reconstruction were included between January 2008 and September 2009. Exclusion criteria were contra-indications for MRI (i.e. claustrophobia, known gadolinium based contrast agent allergy, and an estimated glomular filtration rate below 30 mL/kg/1.73 m^2^). The institutional medical ethics committee of the University Hospital of Maastricht and Maastricht University (METC azM/UM) approved the study and all subjects gave written informed consent before inclusion. All patients underwent preoperative CE-MRA of the abdominal wall and pelvic region to determine the quality and location of the DIEA perforator branches.

### MRI protocol

Examinations were performed using a 1.5-T commercially available system (Intera, Philips Medical Systems, Best, The Netherlands). For signal reception we used a 4-element phased-array parallel imaging-capable body coil with craniocaudal coverage of approximately 25 cm (Philips Medical Systems, Best, The Netherlands). Subjects were imaged in the supine position. The entire examination lasted less than 30 minutes. Imaging parameters for all acquisitions are listed in table 1.

**Table 1:** Acquisition parameters for MRI measurements.

		CE-MRA	
Parameter	TOF	First-pass	Equilibrium-phase
**Scan mode**	Multi 2D	3D	3D
**Technique**	TFE	FFE	FFE
**TR** (ms)	7.20	4.90	12.0
**TE** (ms)	3.20	1.48	1.90
**Flip angle** (°)	50	40	20
**FOV** (mm)	410	400	470
**Voxel dimensions** (acquired) (mm)	1.60×2.34×3.00	1.00×1.36×2.00	0.84×0.84×1.00
**Voxel dimensions** (reconstructed) (mm)	1.60×1.60×3.00	0.78×0.78×1.00	0.84×0.84×1.00
**Number of slices**	100^a^	100^a^	200^a^
**Scan direction**	Axial	Coronal	Coronal
**Parallel imaging acceleration (Factor/direction)**	No	Yes (2/R-L)	Yes (2/R-L)
**NSA**	1	1	1
**Scan duration** (min:sec)	3:37^a^	0:33^a^	4:39^a^

a *The number of slices, and therefore scan duration, varied from subject to subject, depending on the dimensions of the abdomen*.

*TFE, turbo field echo; FFE, fast field echo (gradient echo); FOV, field of view; NSA, number of signal averages*.

#### Survey

A non-enhanced time-of-flight (TOF) scan was acquired to prescribe the imaging volumes of interest for CE-MRA. A turbo field echo (TFE) pulse sequence with a 180° inversion prepulse was used to suppress stationary tissues. One-hundred axial slices were acquired with 3.0-mm slice thickness and 0-mm interslice gap, and an inferiorly concatenated saturation band. The standard quadrature body coil was used for signal transmission and reception. For positioning of the 3D CE-MRA volume a maximum intensity projection (MIP) was generated in 3 orthogonal directions.

#### Contrast

For CE-MRA a fixed dose of 10 mL gadofosveset trisodium (Ablavar®, Lantheus Medical Imaging, Billerica, MA), a blood pool contrast agent, was administered intravenously as a single dose at a speed of 1.0 ml/s in the median cubital vein, using a remote controlled injection system (Medrad Spectris, Indianola, PA). Contrast injection was followed by 20 mL saline flush injected at the same rate. Real time bolus monitoring software (BolusTrak, Philips Medical Systems, Best, The Netherlands) was used to visualize the arrival of the bolus in the abdominal aorta with a refresh rate of proximally 1 frame/sec. Upon first sight of contrast arrival in the abdominal aorta, image acquisition for the first-pass CE-MRA sequence was started. Equilibrium-phase imaging commenced approximately 2 minutes after completion of the first-pass sequence, after allowing systemic contrast equilibration in the arterial and venous blood pool.

### First-pass CE-MRA

First-pass CE-MRA consisted of single station 3D acquisition of the abdominal wall as previously described [20]. Patients were asked to hold their breath as long as possible (inspiration phase) during the acquisition, which lasted approximately 33 seconds.

### Equilibrium-phase high-spatial resolution CE-MRA

A 3D isotropic high spatial resolution equilibrium-phase acquisition of the lower abdomen and pelvic region, comprising both the DIEA and gluteal perforator branches, was performed. As the equilibrium-phase acquisition lasted for approximately 5 minutes, depending on the dimensions of the patient, patients were asked to breathe in a shallow pattern in order to reduce breathing-related motion artifacts as much as possible.

### Image analysis

All equilibrium-phase CE-MRA datasets were analyzed in consensus by a radiologist (BV) and the plastic surgeon (ST) scheduled to perform the DIEP flap dissection and breast reconstruction. A dedicated post-processing workstation was used for image analysis (Vitrea release 4.1.2.0, Vital Images, Minnetonka, MN). Using the original source images as well as coronal and sagittal multiplanar reconstructions (MPR) both first-pass (source) images and equilibrium-phase images were evaluated for (i) image quality; (ii) the location of the single best DIEA perforator at each side of the patient; and (iii) the total number of visualized DIEA perforator branches on each side of the patient. Image quality was assessed on a three-point scale (i.e. excellent quality, diagnostic quality and non-diagnostic quality) and by determination of the vessel-to-noise (VNR) and vessel-to-background (fat tissue) (VBR) ratios of the single best perforator branch [21]. The single best perforator branch was located following the criteria used before by Chernyak et al [6]. The location at which these perforators penetrated the rectus fascia with respect to the center of the umbilicus was noted as x,y-coordinates with the center of the umbilicus being the origin (0,0). The total number of perforator branches visualized by both first-pass and equilibrium-phase imaging were determined within a region extending from 5 cm cranial to 10 cm caudal to the umbilicus.

### Comparison of CE-MRA and intraoperative findings

DIEP flap dissection was performed by a team of three plastic surgeons. Surgeons noted the location of the single best perforator they found during surgery, within the region that was evaluated with MRA. A handheld device ultrasonography and visual/manual inspection were used to identify perforator branches during surgery. After surgery, CE-MRA and intraoperative findings were compared. Data were considered concordant if differences between MRA and intraoperative findings were less than 1 cm in craniocaudal and/or left-right direction.

### Statistical analysis

An independent-samples *t*-test was performed to test the significance of differences in image quality between first-pass and equilibrium-phase CE-MRA and the differences in total number of perforator branches between first-pass and equilibrium-phase CE-MRA and intraoperative findings.

## Results

### Subjects

All included patients underwent MRA without experiencing side effects or adverse events. In twenty-three patients 36 DIEP flaps were successfully dissected. Ten patients underwent unilateral flap dissection, whereas in 13 patients a bilateral flap dissection was performed.

### Diagnostic accuracy of equilibrium-phase high spatial resolution imaging

**Figure 1:**
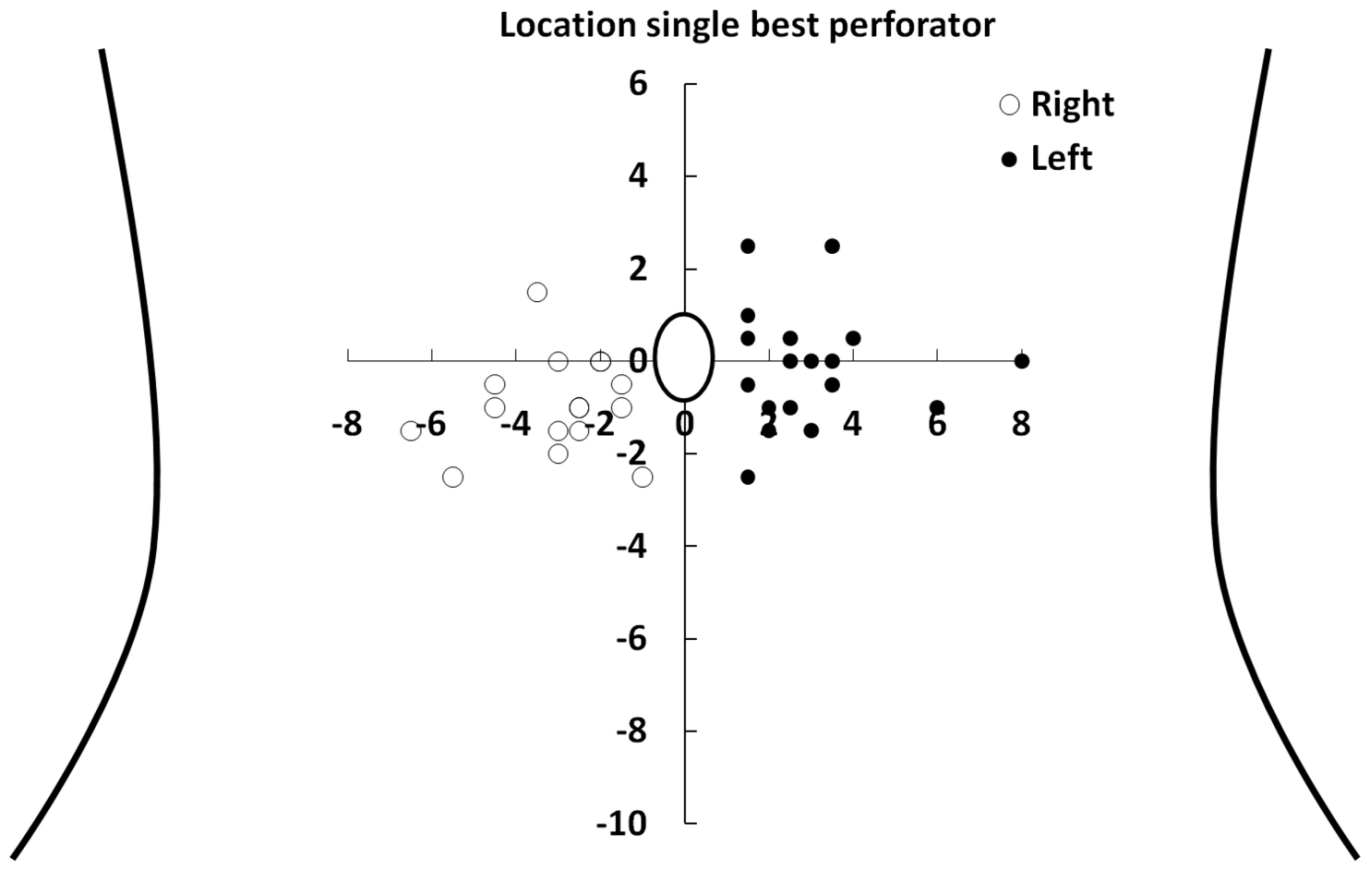
Schematic overview of 36 perforator branches of the deep inferior epigastric artery. Schematic overview of the abdomen and the location of 36 single best perforator branches of the deep inferior epigastric artery found during surgery and with equilibrium-phase high spatial resolution CE-MRA. The x- and y-axis represent the distance (in cm) in respect to the umbilicus.

**Figure 2:**
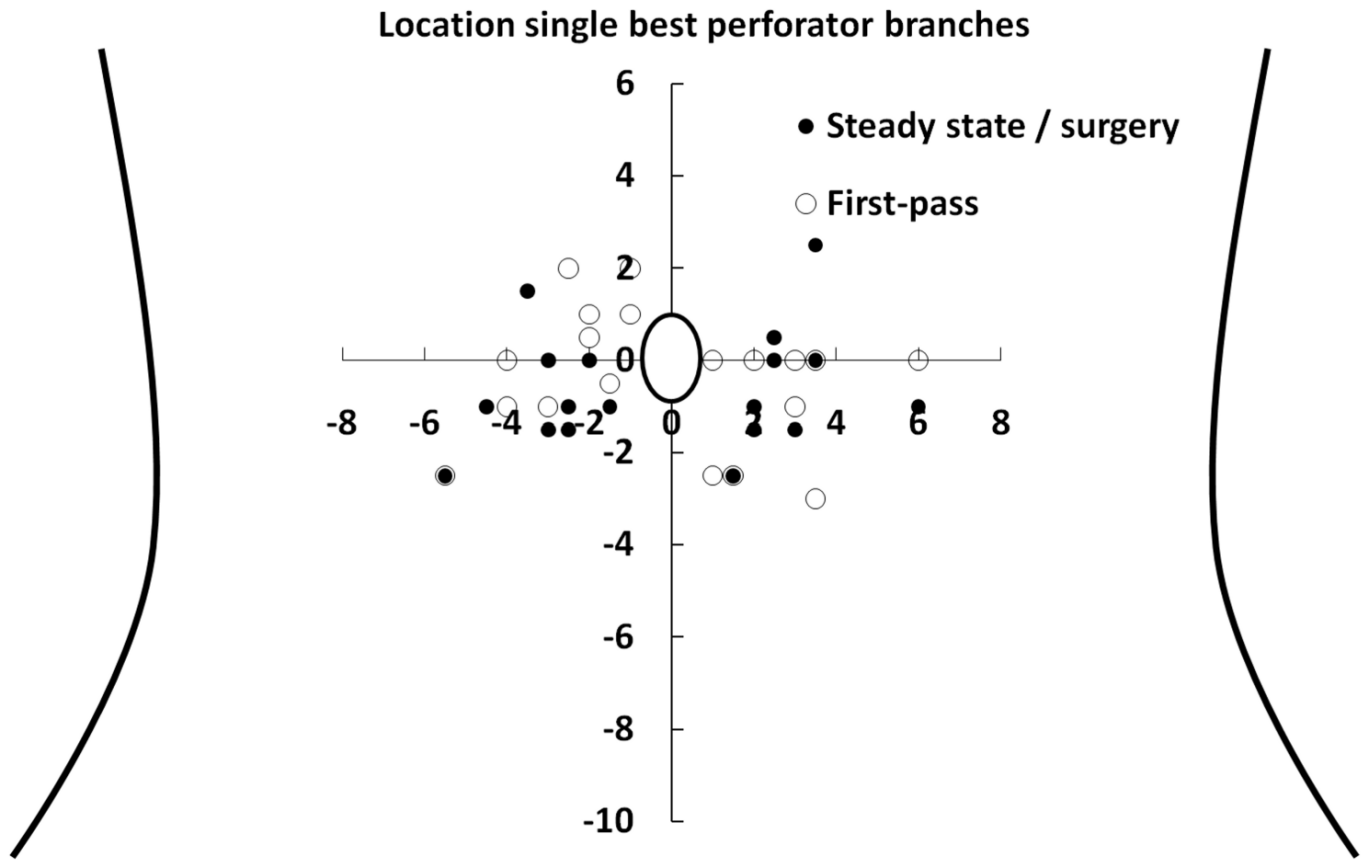
Schematic overview of the best perforator branch of the deep inferior epigastric artery. Schematic overview of the abdomen and the location of the single best perforator branches of the deep inferior epigastric artery found and used during DIEP dissection. The x- and y-axis represent the distance (in cm) in respect to the umbilicus. These perforator branches correlated with equilibrium-phase results for 100%. Only those perforator branches with a first-pass acquisition of diagnostic quality are presented, allowing a 1∶1 comparison between equilibrium-phase high spatial resolution and first-pass CE-MRA.

Equilibrium-phase high spatial resolution acquisitions predicted the location of the single best perforator accurately in all cases, i.e. in 36/36 perforators (100% of the patients). The locations of the perforator branches used for surgery are graphically presented in figure 1. The average location of the single best perforator found during surgery and with equilibrium-phase imaging was located 3.0±1.2 cm (mean ± SD) lateral and 0.6±1.2 cm caudal in respect with the umbilicus (figure 2). There was no significant difference in distance to the umbilicus for left and right sided perforator branches (p = 0.15). We consider now the equilibrium-phase versus first-pass CE-MRA.

### Image quality

**Figure 3:**
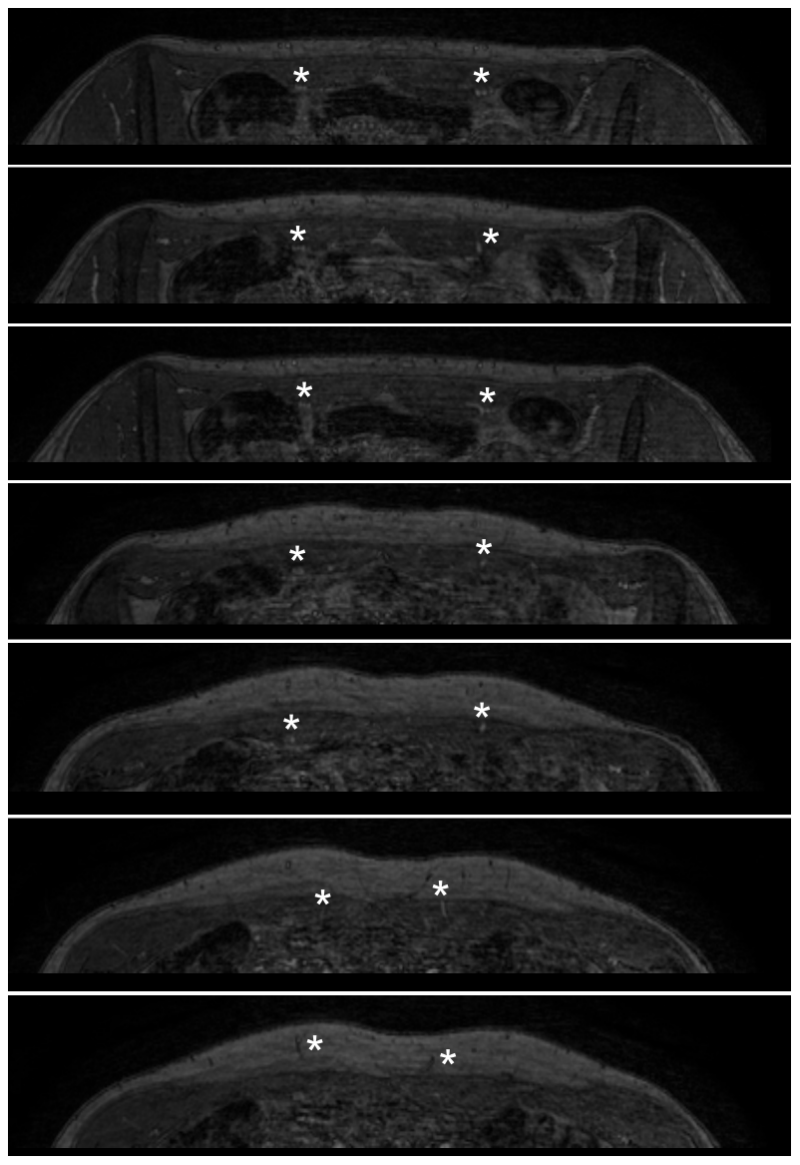
Transverse slices demonstrate the vascular bundle dorsal to the rectus muscle. Transverse source images of equilibrium-phase dataset in the same patient. Images are from caudal (top panel) to cranial (bottom panel), and clearly demonstrate the vascular bundle dorsal to the rectus muscle shortly after branching off the external iliac artery (asterisks in top three panels). The perforating branches can easily be followed when traversing the rectus muscle to the point where they arise in the subcutaneous fat (asterisks in lower 4 panels).

**Figure 4:**
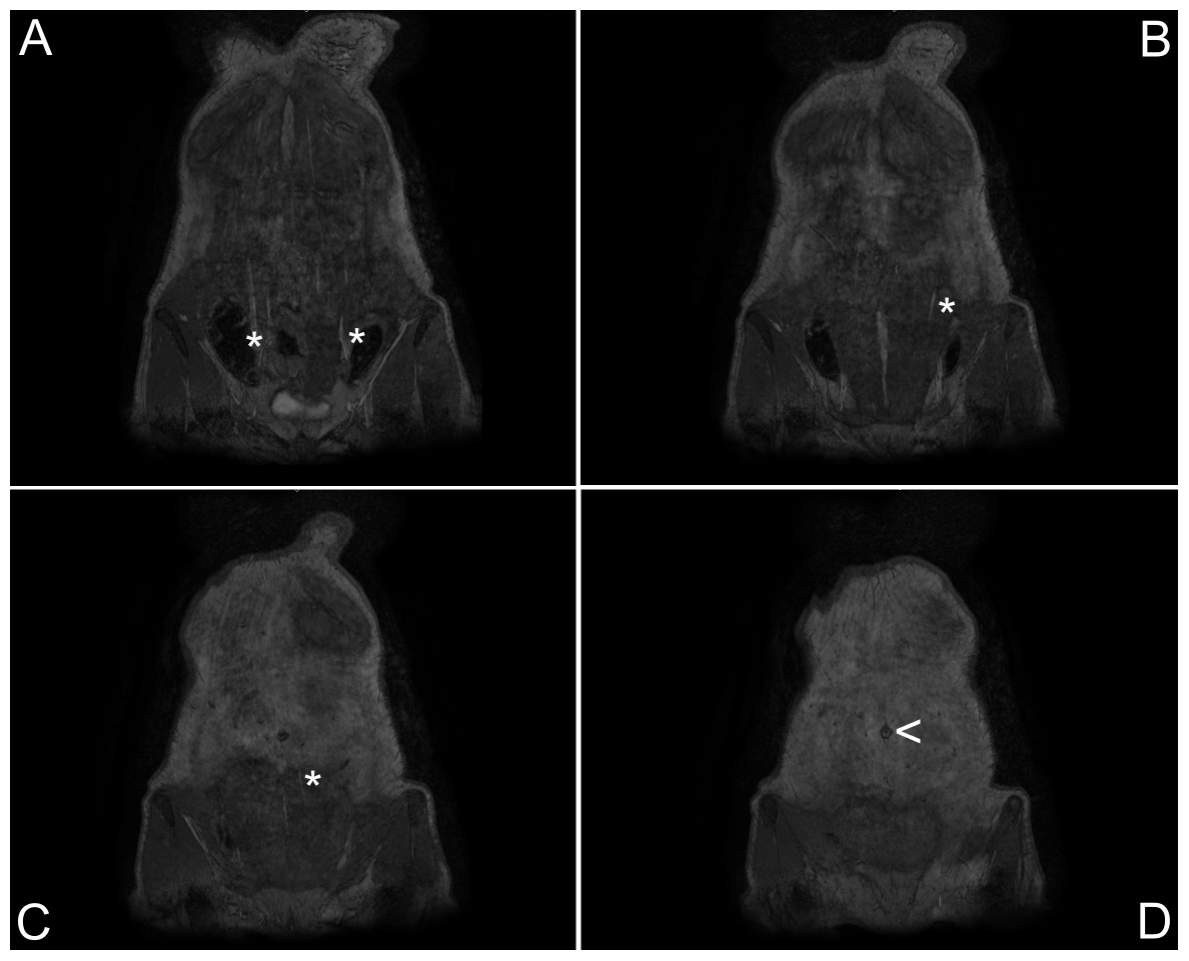
Coronal slices demonstrate the course of perforator branches traversing the rectus muscles. Coronal reformations of equilibrium-phase source images from dorsal (panel A) to ventral (panel D) clearly demonstrate the course of the small perforator branches (asterisks) traversing the rectus muscles. The umbilicus (arrowhead, panel D) is clearly visualized and serves as the reference location for determining the exact point where the perforator branches arise from the muscle. The left side is dominant.

Equilibrium-phase high spatial resolution images were acquired in all patients, whereas because of a timing error first-pass acquisition failed in one patient. Figures 3 and 4 show examples of reconstructed MPR images of an equilibrium-phase acquisition. Image quality results of both equilibrium-phase and first pass acquisitions are presented in table 2. All equilibrium-phase acquisitions were of diagnostic quality, whereas in 10 out of 22 patients the quality of the first-pass images was qualified as non-diagnostic. In those patients it was not possible to identify any perforator branch using the first-pass acquisition. Excellent image quality was obtained in 13 out of 23 patients for equilibrium-phase imaging against only 7 out of 22 in first-pass imaging. Both the signal- and contrast-to-noise ratios were significantly higher for equilibrium-phase imaging in comparison with first-pass acquisitions (p<0.01).

**Table 2:** Image quality in CE-MRA.

	CE-MRA	
Image quality	Equilibrium-phase *(n = 23)*	First-pass *(n = 22)*
Excellent	13 *(57%)*	7 *(32%)*
Diagnostic	10 *(43%)*	5 *(23%)*
Non-diagnostic	0 *(0%)*	10 *(45%)*
VNR (mean ± SD)	16.7±9.1	6.9±4.6^a^
VBR (mean ± SD)	12.0±7.1	3.1±3.5^a^

*VNR, vessel-to-noise ratio; VBR, vessel-to-background ratio*.

a *p<0.01*.

### Perforator branches

The number of perforator branches identified with equilibrium-phase high spatial resolution imaging was significantly higher compared to first-pass imaging (table 3; p<0.01). No significant difference was found in number of perforator branches between the left and right side of patients (p = 0.31 and p = 0.60 in equilibrium-phase and first-pass imaging respectively).

**Table 3:** Number of perforator branches as identified with CE-MRA.

	CE-MRA	
Total number of perforators	First-pass	Equilibrium-phase
Right	3.3±1.4	7.3±2.0^a^
Left	2.8±1.3	7.9±2.0^a^

a *p<0.01*.

### Single best perforator branch

Because of the large number of non-diagnostic first-pass acquisitions, a direct comparison between equilibrium-phase and first-pass imaging was possible in only 12 patients (19 DIEP flaps) (figure 4). In 8 out of 19 single best perforators, the location in first-pass imaging differed more than 1 cm in any direction as compared to equilibrium-phase imaging and surgery.

## Discussion

The accuracy and image quality of contrast-enhanced MR angiography at 1.5T using a blood pool contrast agent in identifying the single best DIEA perforator branch, as desired in the preoperative planning of a deep inferior epigastric perforator flap breast reconstruction procedure, was evaluated, both for equilibrium-phase high spatial resolution and first-pass imaging. Equilibrium-phase high spatial resolution imaging proved 100% accurate in identifying the single best perforator branch compared to intraoperative findings, against only 31% (11 out of 36 perforators) in first-pass imaging. Image quality of equilibrium-phase high spatial resolution imaging was significantly higher compared to first pass imaging.

Preoperative identification of suitable perforator branches of the DIEA is highly valuable because it can shorten anesthesia duration and it makes surgery easier to perform and less traumatic compared to surgery based on ultrasonography [10]. Although it is well known that the best perforators usually lie around the umbilicus, the anatomical variation is very high, which is proven by the results presented in figure 1. This also clarifies why preoperative imaging is really a necessity to decrease dissection time, as blindly localizing the perforator branches with a handheld Doppler device per-operative is quite time consuming, whereas MRA in this study proved 100% accurate in identifying the perforator branches. Moreover the MRA gives informations also about the intramuscular course of the perforator and the connections between perforator and superficial epigastric system, which is also relevant information from a surgical point of view. However, preoperative imaging with CE-MRA is challenging for a number of reasons. First of all, the relatively small caliber of the perforator branches (0.1 to 1 mm when they emerge from the fascia) makes it difficult to find an adequate balance between spatial resolution and acquisition time, especially as contrast-enhanced MR angiography techniques have a limited temporal imaging window due to the relatively fast passage and subsequent wash-out of the contrast agent from the vessels of interest. Secondly, because perforator branches course through the rectus abdominal muscles and the subcutaneous abdominal fat, it can be difficult to acquire sufficient contrast resolution between the perforator branches and the surrounding static tissues. Also, breathing and bowel motion may lead to seriously compromised image quality due to related image artifacts.

Currently, best results with first-pass CE-MRA of the DIEA perforator branches have been achieved at 3.0T, as this field strength allows to optimally balance spatial resolution, contrast resolution and acquisition time [6]. We sought to investigate whether acquisition of equilibrium phase images with higher spatial resolution compared to imaging during first arterial passage resulted in better image quality and improved diagnostic accuracy in identifying the most suitable perforator branch. Diagnostic accuracy of equilibrium-phase high spatial resolution imaging: in this study, we evaluated the diagnostic accuracy of CE-MRA at 1.5 T with gadofosveset trisodium as contrast agent, both for equilibrium-phase high spatial resolution and first-pass imaging. For both acquisitions a 3D gradient echo (FFE) sequence was used. For equilibrium-phase high spatial resolution imaging, there was 100% agreement between intraoperative and MR findings as far as the location of the single best perforator branch was concerned. In all cases there was no more than 1 cm difference in either craniocaudal or left-right direction between equilibrium-phase high spatial resolution MRA and intraoperative findings. This indicates that equilibrium-phase high spatial resolution MR angiography is a very accurate technique for identifying the location of the single best perforator branch in DIEP-procedures and can be a valuable tool for the surgeon to facilitate preoperative planning of the procedure.

### Equilibrium-phase versus first-pass CE-MRA

Whereas equilibrium-phase high spatial resolution imaging was highly accurate, the opposite was true for first-pass imaging. In only 11 out of 36 DIEP flaps, first-pass imaging accurately determined the location of the single best perforator branch. In 10 patients (17 DIEP flaps), first-pass images were not able to identify any perforator branch (non-diagnostic image quality). Besides low accuracy in identifying the single best perforator branches, the total number of perforator branches determined with first-pass imaging was also significantly lower as compared to equilibrium-phase high spatial resolution imaging. The main reason for these poor results was the poor image quality of first-pass imaging.

First pass imaging was only able to identify half the number perforator branches found with steady state imaging. In many cases, these missed perforator branches with first pass imaging turned out to be the single best perforator branch according to steady state imaging. This explains the large mis-match between first pass and steady state imaging.

Image quality in equilibrium-phase imaging was high on the other hand. All equilibrium-phase high spatial resolution examinations were of diagnostic quality and in 13 out of 23 patients the image quality was qualified as excellent (i.e. there were no disturbing artifacts in the region of the single best perforator branch and high signal intensity was found both in the intramuscular and subcutaneous course of the perforator branch), whereas in 10 patients there were minor motion artifacts that did not interfere with the diagnostic accuracy of the exam. In first-pass acquisitions, however, severe motion artifacts due to the inability of patients to sustain a breath hold during acquisition resulted in 10 non-diagnostic examinations. Another important problem with first-pass imaging was the lack of signal in the intramuscular part of perforator branches. Due to this problem, none of the first-pass acquisitions were of excellent image quality. Besides, signal-to-noise and contrast-to-noise ratios for equilibrium-phase imaging were significantly higher as compared to first-pass imaging.

The superior diagnostic accuracy and image quality of equilibrium-phase imaging is probably the result of the relatively high spatial and contrast resolution compared to first-pass imaging. First-pass imaging was able to identify low signal intensity perforator branches within subcutaneous fat tissue, as the low signal intensity of the vessel fascia ensured a strong contrast with the high signal intensity of surrounding fat tissue. However, the intramuscular course of these perforator branches could not be determined due to the lack of intraluminal signal enhancement. However, the exact length and precise intramuscular course of the perforator branch is important as branches that course through muscle over extended lengths are difficult to dissect and associated with more postoperative pain. Equilibrium-phase imaging with a blood pool contrast agent shows that a longer TR and lower flip angle as well as higher spatial resolution results in better contrast resolution (proven by the significant increase in VNR and VBR for equilibrium-phase imaging) and higher sensitivity for identifying small caliber perforator branches respectively.

Motion artifacts caused by breathing reduced image quality both in equilibrium-phase and first-pass imaging. First-pass imaging was performed during a single breath-hold, while patients were freely breathing during equilibrium-phase imaging, as the acquisition time of the equilibrium-phase sequence was approximately 5 minutes. Yet, image distortion due to breathing turned out to be much less severe in equilibrium-phase imaging as compared to first-pass imaging. This is mainly inherent to the imaging technique and the inability of many patients to hold their breath for the requested 33 seconds in first-pass imaging.

### Study limitations

Both equilibrium-phase, but especially first-pass imaging, suffered from motion artifacts, reducing image quality. The influence of breathing may be reduced by imaging patients in prone rather than supine position. Initial findings in our hospital show that abdominal movement is greatly reduced this way, however, drawbacks of this method are distortion of the abdominal wall in prone position and, especially for obese patients, this position is much less comfortable.

Fat suppression might result in an even better contrast resolution and thereby improve image quality. However, use of fat suppression in most cases results in a prolonged acquisition time, which is undesirable for first-pass imaging. For equilibrium-phase imaging image quality already was sufficient, but it is likely that the use of fat suppression will result in even higher VNR and VBR values.

Determining the influence of CE-MRA upon the dissection time of the DIEP flap during surgery was beyond the scope of this study. However, an important next step would be to determine the actual additional value of CE-MRA in both facilitating the preoperative planning of the procedure and the influence upon the dissection time during surgery. This, however, is quite complicated, as many factors are responsible for the dissection time, amongst others the experience and preferences of the surgeon and the between-subject differences in quality and course of the single best perforator branches of the DIEA.

## Conclusion

Equilibrium-phase high spatial resolution CE-MRA of the DIEA perforator branches in the preoperative evaluation of patients undergoing a DIEP flap reconstruction procedure is highly accurate in identifying the single best perforator branch at 1.5T, when using a blood pool contrast agent. Besides accuracy, image quality of equilibrium-phase high spatial resolution imaging proved superior to conventional first-pass CE-MRA.
